# Tumor-Derived Extracellular Vesicles Regulate Cancer Progression in the Tumor Microenvironment

**DOI:** 10.3389/fmolb.2021.796385

**Published:** 2022-01-04

**Authors:** Qianqian Bao, Qianqian Huang, Yunna Chen, Qiang Wang, Ran Sang, Lei Wang, Ying Xie, Weidong Chen

**Affiliations:** ^1^ College of Pharmacy, Anhui University of Chinese Medicine, Hefei, China; ^2^ Anhui Province Key Laboratory of Pharmaceutical Preparation Technology and Application, Hefei, China; ^3^ Anhui Province Key Laboratory of Chinese Medicinal Formula, Hefei, China; ^4^ Bengbu Medical College, Bengbu, China; ^5^ The First Affiliated Hospital of Bengbu Medical College, Bengbu, China; ^6^ State Key Laboratory of Quality Research in Chinese Medicine, Macau University of Science and Technology, Macau, China

**Keywords:** extracellular vesicles, tumor microenvironment, angiogenesis, resistance, immune cells, biomarkers

## Abstract

Extracellular vesicles (EVs) are nanosized particles released by numerous kinds of cells, which are now increasingly considered as essential vehicles of cell-to-cell communication and biomarkers in disease diagnosis and treatment. They contain a variety of biomolecular components, including lipids, proteins and nucleic acids. These functional molecules can be transmitted between tumor cells and other stromal cells such as endothelial cells, fibroblasts and immune cells utilizing EVs. As a result, tumor-derived EVs can deliver molecules to remodel the tumor microenvironment, thereby influencing cancer progression. On the one hand, tumor-derived EVs reprogram functions of endothelial cells, promote cancer-associated fibroblasts transformation, induce resistance to therapy and inhibit the immune response to form a pro-tumorigenic environment. On the other hand, tumor-derived EVs stimulate the immune response to create an anti-tumoral environment. This article focuses on presenting a comprehensive and critical overview of the potential role of tumor-derived EVs-mediated communication in the tumor microenvironment.

## Introduction

Extracellular vesicles (EVs), including exosomes and ectosomes, are nanoscale particles released by nearly all types of cells (Théry et al., 2018). Relying on transferring microRNA (miRNA), long noncoding RNA (lncRNA), messenger RNA (mRNA) and proteins, EVs modulate the functions and phenotypes of target cells (Bayraktar et al., 2017; Choi et al., 2017; Gon et al., 2017). For instance, the delivery of miR-330-3p from plasma cells to ovarian cancer cells by EVs induces a mesenchymal phenotype of ovarian cancers (Yang et al., 2021). In addition, EVs isolated from human vascular endothelial cells contain some cardioprotective proteins, which contribute to promoting human myocardium survival after ischemia-reperfusion injury (Yadid et al., 2020). Vesicular miR-21 derived from tubular epithelial cells stimulates fibroblast and subsequently causes renal fibrosis *in vivo* (Zhao S. et al., 2021). Vesicular lncRNA-SOX2OT from non-small cell lung cancer (NSCLC) cells induces osteoclast differentiation and promotes bone metastasis (Ni et al., 2021).

EVs are closely related to the physical and pathological processes of diseases, especially cancer (Wu et al., 2017; Burnouf et al., 2019; Gamage and Fraser, 2021). Tumor growth requires constant nutrients and oxygen delivered from the vascular network, as they cannot grow above 2 mm^2^ with an inadequate vascular supply (Small et al., 2014). Thereby, angiogenesis, the growth of new blood vessels from the posterior capillary veins and existing capillaries, is vital for tumor progression. EVs mediate communication between tumor cells and endothelial cells, thereby inducing angiogenesis and promoting tumor growth (Wan et al., 2018; Xu et al., 2018). Besides inducing angiogenesis, tumor-derived EVs can also regulate cancer-associated fibroblasts (CAFs) transformation. Since CAFs can remodel the stromal extracellular matrix (ECM) to facilitate tumor cell migration and invasion, CAFs transformation may promote cancer progression. In addition, EVs released from resistant tumor cells have the ability to induce resistance to cancer therapy, which further facilitates tumor progression. Immune cells such as natural killer (NK) cells, macrophages, T cells and B cells can interact with tumor cells via EVs, thereby causing their functions and phenotypes change. Furthermore, crosstalk between tumor-derived EVs and host immune system regulates immune response, thereby influencing cancer progression. Of note, tumor-derived EVs can be isolated from the conditioned medium of cancer cells but also from various body fluids like blood and ascites of cancer patients (Larrea et al., 2016). Due to their cargo diversity and specificity, tumor-derived EVs are promising biomarkers for cancer diagnosis and treatment to reflect the status of parental cancer cells.

## The Biogenesis of EVs

The term EVs is used to describe almost all types of membrane particles secreted from cells. Based on their size and biogenesis, EV subpopulations can be divided into exosomes and ectosomes (Théry et al., 2018). Exosomes are secreted by inward invagination of the plasma membrane (Wolf, 1967; Johnstone et al., 1987). The first invagination of the plasma membrane leads to the generation of an early-sorting endosome that contains fluids, extracellular components and cell surface proteins. The early-sorting endosome undergoes a series of transformations to mature into the late-sorting endosome. The second invagination of the late-sorting endosome results in the formation of multivesicular bodies (MVBs) that contains intraluminal vesicles (ILVs). The MVBs can fuse with the plasma membrane to release exosomes with a size range of 30–150 nm in diameter. The basic mechanisms responsible for exosomes biogenesis have been reported. The endosomal sorting complex required for transport (ESCRT) machinery, containing four protein complexes (ESCRT-0, -I, -II, and -III) along with associated proteins (VTA-1, Alix and VPS4), is closely related to the biogenesis of MVBs and ILVs (Henne et al., 2011). The specific functional components of ESCRT have also been investigated. While the silence of ESCRT-0 and ESCRT-I (HRS) decreases the biogenesis of exosomes, depletion of other ESCRT components exerts no effects or even increases the biogenesis of exosomes (Colombo et al., 2013). ESCRT proteins also play an essential role in specifying the loading of functional cargoes into exosomes. They mediate the sorting of cargo at endosomal plasma and subsequently induce the late-sorting endosomes to release ILVs (later exosomes) with the sorted cargoes. Exosomes biogenesis can operate in an ESCRT-independent manner in some cancer cells, which has been demonstrated by silencing multiple ESCRTs (Stuffers et al., 2009). In addition, some RAB GTPases (RAB27, RAB11, and RAB31) have been found to drive MVBs transport and ILVs biogenesis (Savina et al., 2002; Ostrowski et al., 2010; Wei et al., 2020). For instance, RAB31 can enhance the formation of ILVs and inhibit the degradation of MVBs in an ESCRT-independent manner (Wei et al., 2020). Mechanically, the high level of RAB31 can drive epidermal growth factor receptor into MVBs to generate ILVs and recruit TBC1D2B to prevent MVBs degradation (Wei et al., 2020). However, the upstream of RAB GTPases is not well clarified. Song et al. (2019) found that KIBRA could inhibit the ubiquitination and degradation of RAB27a, thereby contributing to exosomes biogenesis (Song et al., 2019). Phospholipase D2 and its product phosphatidic acid are involved in ILVs biogenesis and exosomes release (Egea-Jimenez and Zimmermann, 2018). Notably, ESCRT-dependent pathways and ESCRT-independent pathways can also jointly drive exosomes biogenesis. Syndecan-syntenin complexes bind ESCRT-I and ESCRT-III via Alix, leading to enhanced ILVs biogenesis (Baietti et al., 2012). Moreover, tyrosine phosphatase Shp2 has been found to inhibit exosomes biogenesis via dephosphorylating syntenin (Zhang Y. et al., 2021). CD63, belonging to tetraspanin family, is associated with sorting cargoes into exosomes (Theos et al., 2006; van Niel et al., 2011).

Ectosomes with a size range of 50–1,000 nm in diameter are released by shedding or outward budding of the plasma membrane. This process is driven by translocating phosphatidylserine to the outer-membrane leaflet (Zwaal and Schroit, 1997). The mechanisms involved in the biogenesis of ectosomes have been studied. Inhibiting VPS4 is shown to impair ectosomes release, suggesting that ESCRT-III is also required for ectosomes biogenesis (Mathieu et al., 2019). Small GTPase RhoA, an essential regulator of actin cytoskeletal remodeling, is closely related to ectosomes biogenesis in different tumor cells (Li et al., 2012). Moreover, RHO-associated protein kinase (ROCK) has been revealed to mediate the function of RhoA in ectosomes biogenesis. Thus, inhibition of ROCK-1 and ROCK-2 by the small molecule Y-27632 can decrease ectosomes biogenesis (Li et al., 2012). Ectosomes are rich in cholesterol, and knockdown of cholesterol can inhibit ectosomes biogenesis (Del Conde et al., 2005). In addition, nSMAse participates in shedding budding of the plasma membrane, and hence controls ectosomes biogenesis (Menck et al., 2017).

## Tumor Microenvironment and Tumor-Derived EVs

**FIGURE 1:**
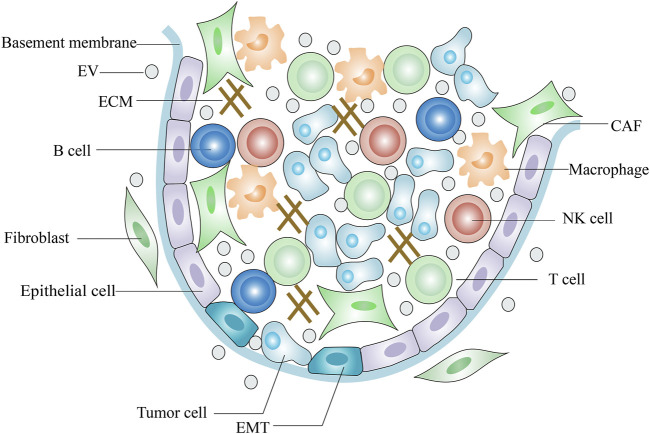
Scheme of tumor microenvironment.

The TME (tumor microenvironment) comprises tumor cells, endothelial cells, fibroblasts, and immune cells as well as extracellular components such as ECM, cytokines and growth factors (Lv et al., 2012; Jin and Jin., 2020) (Figure 1). The ECM is a highly dynamic three-dimensional network composed of plenty of fibrous proteins and glycoproteins (Mouw et al., 2014). Tumors often exhibit ECM deposition and degradation, and this dysregulation state supports tumorigenesis and metastasis and induces angiogenesis (Sükei et al., 2021). Cytokines are small molecular polypeptides or proteins that serve as immunomodulatory effectors. Overproduction of IL-6 by tumor cells activates STAT-3, a key transcription factor central to immune escape and it is an important regulator in the crosstalk between tumor cells and TME (Lokau et al., 2019). IL-10 is an anti-inflammatory cytokine and its serum levels are negatively related to the tumor prognosis (Pasvenskaite et al., 2021). On the other hand, IL-10 exerts anti-tumor activity by enhancing the immune-stimulatory effect of CD8^+^ T cell (Naing et al., 2018). Some growth factors in the TME inhibit normal stromal cells proliferation and promote tumor cells metastasis. Transforming growth factor-β (TGF-β), fibroblast growth factor (FGF) and vascular endothelial growth factor (VEGF) form a pro-tumorigenic environment that fosters tumor cell survival, progression and metastasis and directs abnormal vessel growth (Zhou X. et al., 2018; Shang et al., 2020; Zheng et al., 2021). However, TGF-β also shows anti-oncogenic properties in carcinogenesis. TGF-β has been reported to inhibit tumor cell proliferation and induce apoptosis in the early stages of carcinogenesis (Inman, 2011).

As a means of communication between tumor cells and the microenvironment, EVs play an essential role in remodeling the local microenvironment (Milane et al., 2015). Numerous studies have revealed that EVs released by tumor cells contain a variety of biomolecular components, including lipids, proteins and nucleic acids (Taylor et al., 2011; Mathivanan et al., 2012; Agudiez et al., 2020). Especially, those nucleic acids components such as miRNAs and lncRNAs may mediate the formation of a protumoral or an anti-tumoral soil in the microenvironment, thereby influencing tumor progression. Interestingly, hypoxic or metastatic status of tumors appears to a strong force in sorting the loading of composition into EVs, which affects functions of tumor-derived EVs in the TME (Kucharzewska et al., 2013; Yokoi et al., 2017; Chen et al., 2018). Hypoxia is a common feature in most malignant tumors. In hypoxic microenvironment, tumor cells drive glucose mainly into lactate to meet the energy requirements. This phenomenon, called the Warburg effect, is one of the cellular mechanisms by which cancer cells adapt to hypoxic microenvironment and enhance survival (Parks et al., 2017). PKM2, which plays a key role in the Warburg effect, is the enhancer of anaerobic glycolysis. Hypoxic NSCLC cell derived-EVs promote PKM2-dependent glycolysis and subsequently produce metabolites to eliminate ROS, thereby inhibiting tumor apoptosis and promoting tumor growth (Wang et al., 2021). EVs derived from breast cancer enhance the ability of CAFs in response to different metabolic environment by activating MYC signaling pathway in stromal cells resulting in rapid tumor growth (Yan et al., 2018). Moreover, EVs released from Lewis lung carcinoma can induce immunosuppressive macrophages by NF-κB-mediated metabolism reprogramming, leading to tumor metastasis (Morrissey et al., 2021).

## Regulation of Protumoral Functions of Endothelial Cells by Tumor-Derived EVs

Tumor-derived EVs are thought to regulate protumoral functions of endothelial cells in numerous types of cancers including hepatocellular carcinoma (HCC) (Lin et al., 2018), colorectal cancer (Huang and Feng, 2017; Zeng et al., 2018; He et al., 2021), cervical cancer (Wu et al., 2019), nasopharyngeal carcinoma (Bao et al., 2018; Tian et al., 2021; Zhang K. et al., 2021), glioma (Ma et al., 2017; Wang Z.-F. et al., 2019), and lung cancer (Hsu et al., 2017). As EVs can be internalized by an endocytic-like process, they may deliver regulatory biomolecules into vascular endothelial cells. Thus, tumor cells can participate in the regulation of endothelial cell proliferation, migration, sprouting, branching, as well as tubular-like structure formation by secreting EVs (Zhuang et al., 2012; French et al., 2017).

Some miRNAs, lncRNAs and proteins delivered by EVs have been reported to participate in regulating protumoral functions of endothelial cells (Table 1). miR-210 enriched in EVs of malignant tumors may promote tubular-like structure formation of endothelial cells, leading to pro-angiogenic activities and rapid tumor growth. In HCC, abundant miR-210 can be packed into EVs and transferred to endothelial cells (Lin et al., 2015; Lin et al., 2018). After taking up by human umbilical vein endothelial cells (HUVECs), miR-210 stimulates angiogenesis via down-regulating the expression of SMAD4 and STAT6 (Lin et al., 2018). Vesicular miR-21-5p from colorectal cancer decreases Krev interaction trapped protein 1 expression to activate β-catenin signaling pathway and promote the expression of angiogenesis-related factors like VEGFA, thereby stimulating vascular permeability and angiogenesis (He et al., 2021). In addition, cervical cancer-derived vesicular miR-221-3P promotes angiogenesis by inhibiting the expression of thrombospondin-2 in HUVECs, which consequently enhances tumor growth *in vivo* (Wu et al., 2019). miR-144 is a key angiogenesis inducer for neo-angiogenesis in nasopharyngeal carcinoma (Tian et al., 2021). Vesicular miR-144 suppresses FBXW7 and increases hypoxia-inducible factor-1α (HIF-1α) and VEGFA in recipient cells, which consequently promotes endothelial cells migration and invasion (Tian et al., 2021). Moreover, the transfer of high mobility group box 3 (HMGB3) from nasopharyngeal carcinoma cells to endothelial cells via EVs induces angiogenesis (Zhang K. et al., 2021). Interestingly, neo-angiogenesis in nasopharyngeal carcinoma facilitates the formation of pre-metastatic niches, which further causes tumor metastasis (Zhang K. et al., 2021). Vesicular miR-26a from glioma down-regulates phosphatase and tensin homolog (PTEN) expression to stimulate PI3k/AKT signaling, thereby contributing to the proliferation of human brain microvascular endothelial cells (HBMECs) (Wang Z.-F. et al., 2019). Ma et al. (2017) believe that the delivery of lncRNA HOTAIR from glioma cancer cells to HBMECs *via* EVs up-regulates the level of pro-angiogenic factor VEGFA.

**TABLE 1:** Role of tumor-derived EVs in angiogenesis.

Cargoes	Cancer types	Mechanisms	References
miR-210	HCC	SMAD4 and STAT6↓	Lin et al. (2015), Lin et al. (2018)
miR-21-5P	Colorectal cancer	Krev interaction trapped protein 1↓; β-catenin signaling pathway, VEGFA and Ccnd1↑	He et al. (2021)
miR-221-3p	Cervical cancer	Thrombospondin-2↓	Wu et al. (2019)
miR-144	Nasopharyngeal carcinoma	FBXW7↓; HIF-1α and VEGFA↑	Tian et al. (2021)
HMGB3	Nasopharyngeal carcinoma	Unknown	Zhang et al. (2021a)
miR-26a	Glioma	PTEN↓; PI3k/Akt signaling pathway↑	Wang et al. (2019c)
LncRNA HOTAIR	Glioma	VEGFA↑	Ma et al. (2017)
Wnt4	Colorectal cancer	Wnt/β-catenin signaling pathway↑	Huang and Feng (2017), Yamada (2017)
miR-23a	Lung cancer	Prolyl hydroxylase 1/2↓; HIF-1α↑	Hsu et al. (2017)
miR-23a	Nasopharyngeal carcinoma	Testis-specific gene antigen↓	Bao et al. (2018)
miR-25-3p	Colorectal cancer	Krüppel-like factor 2, Krüppel-like factor 4, occludin, zonula occludens-1 and Claudin5↓; VEGFR2↑	Zeng et al. (2018)

Symbols: ↑, up-regulation; ↓, down-regulation.

It is well-known that disordered vascular distribution and abnormal vascular structure lead to specific hypoxia in many solid tumors. In turn, tumor-derived EVs secreted under hypoxic conditions induce proliferation and migration of endothelial cells, thereby enhancing angiogenesis and tumor growth. For example, EVs isolated from colorectal cancer cells under hypoxia conditions show a more potent pro-angiogenic effect as compared with that from colorectal cancer cells under normoxia conditions (Huang and Feng, 2017). The reason for this phenomenon may be that Wnt4 is highly enriched in hypoxic colorectal cancer-derived EVs, and the increased Wnt4 stimulates the β-catenin signaling pathway in endothelial cells (Yamada, 2017). Similarly, lung cancer-derived vesicular miR-23a under hypoxia conditions enhances the production of HIF-1α in endothelial cells via inhibiting the expression of prolyl hydroxylase 1/2, thereby directly promoting angiogenesis and tumor growth (Hsu et al., 2017).

On the other hand, the emerging evidence has shown that the contents of tumor-derived EVs may be enriched at the metastatic stage during cancer development, and those increased contents can be delivered to endothelial cells to exert biological roles. For example, miR-23a is shown to be significantly higher in nasopharyngeal carcinoma tissues with metastasis than those without metastasis, and its level is associated with angiogenesis (Bao et al., 2018). Furthermore, the molecular mechanism for vesicular miR-23a-mediated angiogenesis may be related to testis-specific gene antigen (Bao et al., 2018). Metastasis-induced vesicular miR-25-3p promotes vascular permeability and angiogenesis, leading to the formation of pre-metastatic niches (Zeng et al., 2018). Mechanically, vesicular miR-25-3p in colorectal cancer can silence Krüppel-like factor 2 and krüppel-like factor 4, thereby enhancing the expression of vascular endothelial growth factor receptor 2 (VEGFR2) and inhibiting the expression of occludin, zonula occludens-1 and Claudin5 (Zeng et al., 2018).

Tumor-derived EVs play an important role in inducing angiogenesis. Similarly, EVs derived from endothelial cell and perivascular cell are also a key player in tumor progression. Anti-angiogenic therapies are thought to improve the prognosis of tumor patients by inhibiting tumor vascularization. However, the outcomes of anti-angiogenic therapies are not ideal for most patients. EVs released from endothelial cells treated with vandetanib enrich VEGF, thus promoting angiogenesis and tumor growth *in vivo* (Zeng et al., 2019). In addition, EVs released from perivascular cell trigger endothelial progenitor cells recruitment after anti-angiogenic therapy cessation, which contributes to blood vessel regrowth and rapid tumor growth (Huang et al., 2021). Mechanically, Gas6-containing perivascular cell-derived EVs activate Axl signaling and subsequently promote tumor revascularization (Huang et al., 2021).

## Regulation of Protumoral Functions of CAFs by Tumor-Derived EVs

**FIGURE 2:**
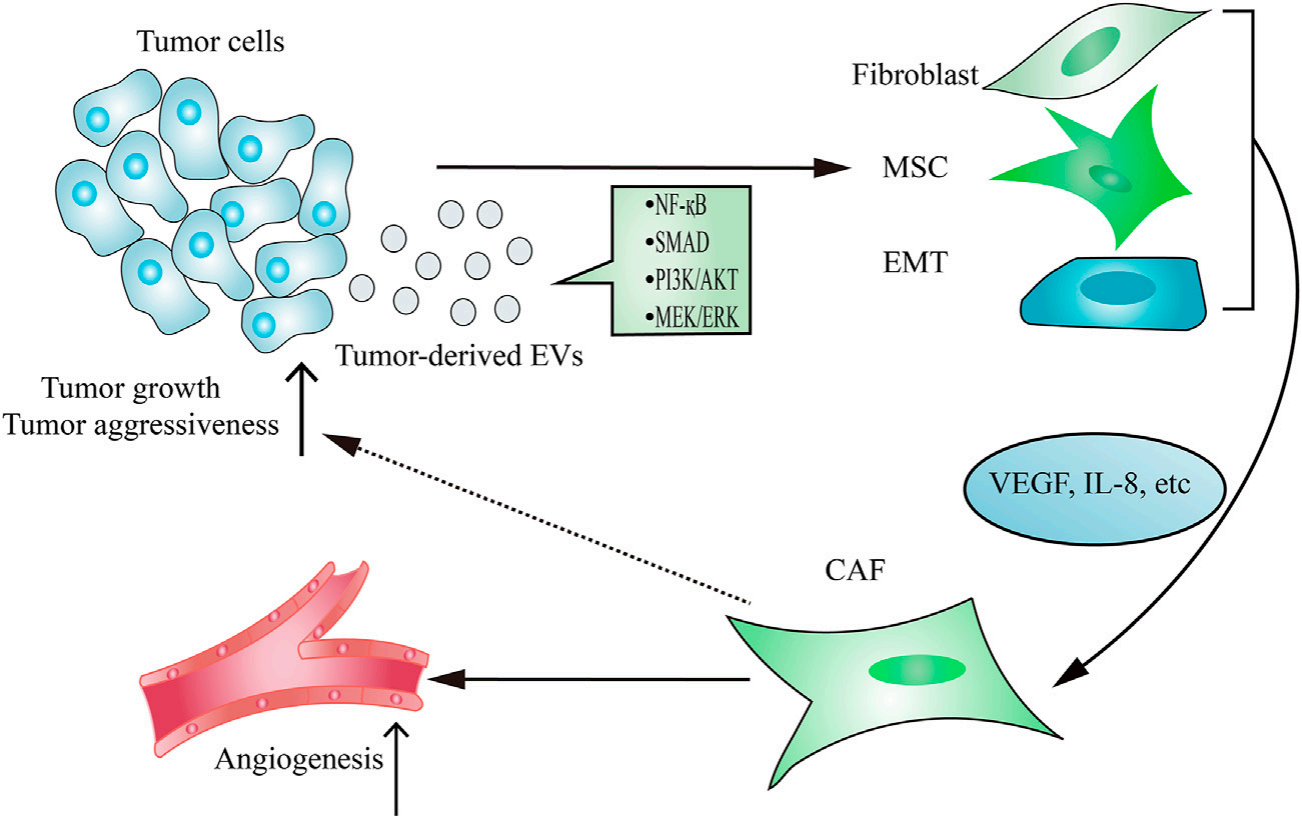
Tumor-derived EVs regulate CAFs transformation. The delivery of functional signaling factors from tumor cells to fibroblasts, MSCs or EMT contributes to CAFs transformation, which consequently promotes tumor growth and aggressiveness and induces angiogenesis.

As the main contributor to remodel tumor stroma, CAFs are often transformed from resident fibroblasts, mesenchymal stem cells (MSCs) and epithelial-to-mesenchymal transition (EMT) cells after taking up tumor-derived EVs (Figure 2). The active CAFs may enhance angiogenesis and metastasis, thereby contributing to establishing a tumor-promoting environment. Hodgkin lymphoma-derived EVs transform normal fibroblasts into pathological CAFs utilizing the NF-κB signaling pathway, which leads to the release of neo-angiogenesis factors (Dörsam et al., 2018). Notably, many studies have shown that the delivery of functional biomolecules plays a vital role in regulating CAFs transformation (Paggetti et al., 2015; Fang et al., 2018; Giusti et al., 2018; Yang et al., 2018; Wang J. et al., 2018; Zhou Y. et al., 2018). For instance, EVs released from chronic lymphocytic leukemia cells induce fibroblasts transformed to CAFs by the enrichment of some regulatory proteins and miRNAs from parental cells, which consequently causes rapid tumor growth (Paggetti et al., 2015). In addition to regulating the transformation of fibroblasts into a CAF phenotype, tumor-derived EVs have also been demonstrated to play an important role in inducing the transition of MSCs into CAFs. EVs isolated from breast cancer stimulate SMAD-mediated pathway and subsequently increase CAFs marker expression in MSCs, which consequently enhances angiogenesis and metastasis (Cho et al., 2012). Moreover, tumor cells-derived EVs are capable of regulating the transformation of pericytes into a pathological CAFs phenotype. Relying on releasing EVs, gastric cancer cells promote pericytes proliferation and migration, and induce pericytes transformed into CAFs (Ning et al., 2018). Mechanically, gastric cancer cells-derived EVs stimulate PI3k/AKT and MEK/ERK pathways, leading to the up-regulated expression of CAFs markers (Ning et al., 2018).

Due to the emerging evidence indicates EVs isolated from tumor cells response to hypoxia, many researchers have investigated the potential of tumor-derived EVs under hypoxia conditions in CAFs transformation (Kucharzewska et al., 2013; Wang et al., 2014; Ramteke et al., 2015). It has been reported that EVs secreted from prostate cancer cells under hypoxia conditions promote CAFs transformation and tumor aggressiveness (Ramteke et al., 2015). Interestingly, EVs derived from tumor cells can not only enrich some proteins, but also load some specific proteins that may induce tumor-promoting microenvironment under hypoxia conditions (Ramteke et al., 2015). This finding suggests that unique components loaded in hypoxia tumor-derived EVs may be helpful to CAFs transformation and tumor progression.

On the other hand, tumor-derived EVs appear to be enriched during fibroblasts reprogramming may be a reaction to the high-metastatic tumor state. Additionally, the functional contents of EVs can also be related to the metastatic status of HCC cells (Fang et al., 2018). The amount of EVs is much higher in high-metastatic cancer cells than that in low-metastatic cancer cells (Fang et al., 2018). This further enhances the regulatory effect of high-metastatic tumor cell-derived EVs on CAFs transformation. Mechanically, elevated miR-1247-3p in EVs isolated from high-metastatic tumor cells that promotes CAFs transformation via inhibiting B4GALT3 expression (Fang et al., 2018).

Tumor-derived EVs play an important role in CAFs transformation, in turn, CAF-derived EVs participate in tumorigenesis. For instance, vesicular miR-92a-3p from CAFs induce EMT, chemoresistance and cancer stemness in colorectal cancer by activating Wnt/β-catenin signaling pathway (Hu et al., 2019). EVs released from CAFs enrich miR-196a by activating heterogeneous nuclear ribonucleoprotein A1, leading to decreased CDKN1B and ING5 in recipient head and neck cancer cancer cells, and ultimately result in enhanced cisplatin resistance and metastasis (Qin et al., 2019). Moreover, ubiquitin-specific protease 7 has been found to inhibit heterogeneous nuclear ribonucleoprotein A1 ubiquitination in CAFs (Zhang et al., 2020). miR-522-bearing EVs released from CAFs inhibit arachidonate lipoxygenase 15 expression and lipid peroxides accumulation, leading to enhanced chemoresistance in gastric cancer (Zhang et al., 2020). Thus, depletion of CAF-derived EVs causes improved chemosensitivity.

## Regulation of Resistant Phenotype of Sensitive Cancer Cells by Tumor-Derived EVs

Emerging studies have confirmed that tumor-derived EVs play a vital role in the resistance of tumor cells to cancer therapy, including chemotherapy and radiotherapy (Table 2). Some drug-resistant tumor cells have the ability to confer a drug-resistant phenotype upon sensitive cells in an EVs-dependent manner. This may be due to EVs’ ability to mediate the transfer of miRNA, lncRNA and proteins associated with drug resistance to recipient cells (Corrado et al., 2013). For instance, paclitaxel-resistant gastric cancer cells have been reported to be rich in miR-155-5p (Wang M. et al., 2019). miR-155-5p can be delivered from resistant cancer cells to sensitive cells by EVs, thereby increasing the expression level of miR-155-5p in recipient cells. The increased miR-155-5p confers paclitaxel resistance and induces EMT in gastric cancer cells via inhibiting the expression of GATA3 and TP53INP1 (Wang M. et al., 2019). Similarly, miR-423-5p-bearing EVs induce the transformation of breast cancer cells from sensitive cells to cisplatin-resistant cells (Wang B. et al., 2019). While the above examples show that drug-resistant tumor-derived EVs can disseminate drug resistance via transferring increased miRNAs to recipient cells, it appears that EVs can also induce drug-resistance by decreased miRNAs. EVs released from cisplatin-resistant lung cancer cells down-regulate a total of 11 miRNAs, in which miR-100-5p is the most significantly down-regulated miRNA (Qin et al., 2017). The down-regulated miR-100-5p modulates the expression of the mammalian target of rapamycin in recipient cells, which induces a chemo-resistant phenotype upon NSCLC cells (Qin et al., 2017).

**TABLE 2:** Role of tumor-derived EVs in therapy resistance.

Cargoes	Cancer types	Functions	Mechanisms	References
miR-155-5p	Gastric cancer	Paclitaxel resistance and EMT↑	GATA3 and TP53INP1↓	Wang et al. (2019b)
miR-423-5p	Breast cancer	Cisplatin resistance, breast cancer cells proliferation and migration↑	P-glycoprotein↑	Wang et al. (2019a)
miR-100-5p	Lung cancer	Cisplatin resistance↑	Mammalian target of rapamycin↑	Qin et al. (2017)
LncRNA H19	NSCLC	Gefitinib resistance↑	Unknown	Lei et al. (2018)
LncRNA-SNHG14	Breast cancer	Trastuzumab resistance↑	Bcl-2/Bax apoptosis signaling pathway↑	Dong et al. (2018)
TrpC5	Breast cancer	Adriamycin resistance↑	P-glycoprotein↑	Ma et al. (2014)
PKM2	NSCLC	Cisplatin resistance and NSCLC cells proliferation↑	CAFs transformation↑	Wang et al. (2021)
Annexin A6	Triple-negative breast cancer	Gemcitabine resistance↑	Epidermal growth factor receptor↓	Li et al. (2021)
Unknown	Squamous head and neck cancer	Radio-resistance and Squamous head and neck cancer cells proliferation↑	Repair of damaged DNA content↑	Mutschelknaus et al. (2016)
miR-301a	Glioma	Radio-resistance↑	TCEAL7↓; Wnt/β-catenin signaling pathway↑	Yue et al. (2019)
Anaplastic lymphoma kinase	NSCLC	Anaplastic lymphoma kinase inhibitors resistance, Ceritinib resistance and tumor growth↑	AKT, STAT3 and ERK signaling pathways↑	Wu et al. (2018)

Symbols: ↑, up-regulation; ↓, down-regulation.

The expression of lncRNA H19 is up-regulated within EVs from gefitinib-resistant NSCLC cells (Lei et al., 2018). Vesicular lncRNA H19 can be transported to sensitive cells to induce gefitinib resistance (Lei et al., 2018). EVs isolated from trastuzumab-resistant HER2^+^ breast cancer increase the level of lncRNA-SNHG14, which can induce a chemo-resistant phenotype upon sensitive tumor cells (Dong et al., 2018). Besides mediating the transfer of miRNA or lncRNA to induce drug resistance, EVs have also been shown to deliver proteins to target cells to disseminate resistance. For instance, EVs released from patients with a poor response to chemotherapy up-regulate the expression of transient receptor potential channel 5 (TrpC5) (Ma et al., 2014). Relying on EVs, the increased TrpC5 can enter sensitive breast cancer cells to disseminate resistance. In addition, PKM2-bearing EVs from cisplatin-resistant tumor cells induce a chemo-resistant phenotype upon NSCLC cells by reprogramming CAFs transformation (Wang et al., 2021). Recently, vesicular transfer of annexin A6 has been found to confer gemcitabine-resistance in sensitive triple-negative breast cancer cells via suppressing and degrading of epidermal growth factor receptor (Li et al., 2021).

Additionally, the involvement of EVs in the resistance of tumor cells to radiotherapy has been reported. Early data demonstrated that the protein composition of tumor-derived EVs might be changed when exposed to radiation (Jelonek et al., 2015). Apart from affecting the composition of EVs, radiation has also been shown to affect the functions of EVs on target cells. EVs isolated from irradiated squamous head and neck cancer cells can confer radio-resistance in recipient cells via repairing damaged DNA content (Mutschelknaus et al., 2016). EVs isolated from hypoxic glioma are rich in miR-301a, which is associated with the resistance of tumor cells to radiotherapy (Yue et al., 2019). Mechanically, miR-301a-bearing EVs directly target TCEAL7 gene to induce radio-resistance in glioma cells and this effect can be reversed by inhibiting the Wnt/β-catenin pathway (Yue et al., 2019). Similarly, EVs released from irradiated cells can also reduce the sensitivity of recipient cells to the drug (Wu et al., 2018). Mechanically, EVs released from NSCLC cells can induce anaplastic lymphoma kinase inhibitors-resistant or Ceritinib-resistant phenotype upon target tumor cells via stimulating AKT, STAT3 and ERK pathways (Wu et al., 2018).

## Tumor-Derived EVs Modulate the Immune System

**FIGURE 3:**
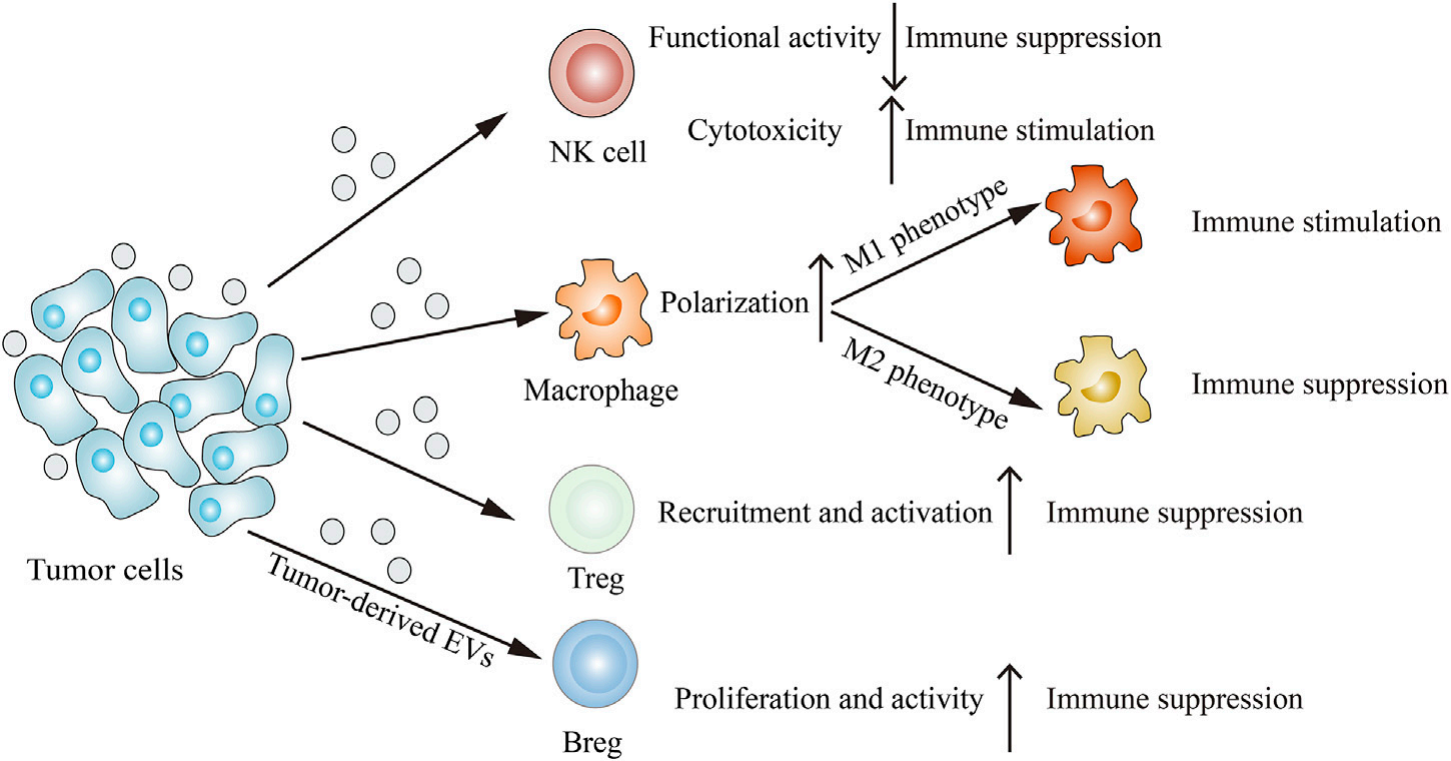
Tumor-derived EVs modulate the immune system. Tumor-derived EVs can suppress NK cells functional activity, induce M2 macrophage polarization, recruit Tregs and proliferate Bregs, thus inhibiting immune response. On the other hand, tumor-derived EVs can also enhance NK cells cytotoxicity and induce M1 macrophage polarization, thereby stimulating immune response.

Immune cells such as NK cells, macrophages, T cells and B cells can interact with tumor cells, resulting in their functions and phenotypes changes. The emerging report reveals that tumor-derived EVs are involved in remodeling the tumor immune microenvironment (Figure 3). Through releasing EVs, tumor cells can deliver immune-inhibitory and immune-stimulatory signaling biomolecular components to the tumor immune microenvironment, thus creating a protumoral or an anti-tumoral soil to influence cancer progression (Whiteside, 2016).

### NK Cells

NK cells, which play a key role in cancer immunotherapy, are the important subset of innate immune cells. Early research demonstrated that NK cell activity could be inhibited by breast cancer-derived EVs, which resulted in the accelerated growth of xenograft tumors (Liu et al., 2006). Researchers isolated NK cells from the spleens of BALB/c mice that had been pretreated with purified breast cancer-derived EVs and determined NK cell activity by the chromium release assays (Liu et al., 2006). Trials have shown that NK cell cytolytic activity was inhibited in mice by EVs released from TS/A tumor cells (Liu et al., 2006). Further study has demonstrated that pretreated mice with TS/A tumor cells-derived EVs would lead to a significant decrease in the total number and percentages of NK cells (Liu et al., 2006). Given the accumulating evidence for the role of EVs isolated from tumor cells in response to hypoxia, some researchers have investigated the potential of tumor-derived EVs under hypoxia conditions in reprogramming functions of NK cells. For instance, EVs secreted from tumor cells under hypoxia conditions show a more potent ability to impair the cytotoxicity of NK cells as compared with that from tumor cells under normoxia conditions (Berchem et al., 2016). In addition, the expression levels of functional activity markers such as CD107a and IFN-γ in NK cells pretreated with hypoxic tumor-derived EVs are significantly lower than those in NK cells pretreated with normoxic tumor-derived EVs (Berchem et al., 2016). This could be in part explained by the abundance of miR-23a in hypoxic tumor cells-derived EVs that could function as an additional immunosuppressive activator by directly targeting CD107a in NK cells (Berchem et al., 2016). On the other hand, EVs secreted from pancreatic cancer cells at the high-metastatic state also appear to down-regulate the expression of CD107a and IFN-γ in NK cells (Zhao et al., 2019). Furthermore, EVs isolated from pancreatic cancer patients contain abundant TGF-β1, which can attenuate CD107a and IFN-γ expression in NK cells (Zhao et al., 2019). Mechanically, TGF-β1-bearing EVs activate the TGF β-Smad2/3 pathway in NK cells to impair NK cell-mediated cytotoxicity (Zhao et al., 2019). Apart from inhibiting the cytotoxic activity of NK cells, tumor-derived EVs have also been shown to act as an inducer to stimulate effective NK cell anti-tumor response. For example, EVs isolated from resistant anti-cancer drug-treated HCC cells are able to stimulate the suppressive effects of NK cells on tumor cell proliferation (Lv et al., 2012). The reason for this phenomenon may be that EVs released from HCC cells treated with resistant anti-cancer drugs contain abundant heat shock proteins (HSPs), including HSP60, HSP70 and HSP90 (Lv et al., 2012). Notably, those resistant anti-cancer drugs promote HSP-bearing EVs release, thereby contributing to activating the cytotoxic response of NK cells (Lv et al., 2012).

### Macrophages

Macrophages, as one part of innate immune systems, can be affected by many factors to switch their phenotype. Activated macrophages are commonly classified into two phenotypes, classical activation (M1) macrophages and alternative activation (M2) macrophages. M1 macrophages secrete pro-inflammatory cytokines to induce tumoricidal activity, while M2 macrophages secrete anti-inflammatory cytokines to promote tumorigenesis. Tumor-associated macrophages (TAMs) are the main immune cell population in TME, which can be educated by various tumor cells and display an M2-like phenotype to promote the development and progression of tumors. Nowadays, tumor-derived EVs are described as containing a variety of functional components and are now emerging as a key regulator of macrophage polarization. Those components, such as miRNA, lncRNAs and proteins, can be transferred to macrophages via EVs to switch their phenotype.

Epithelial ovarian cancer-derived EVs can induce macrophages to secrete anti-inflammatory cytokine IL-10, leading to enhanced tumor growth and metastasis (Ying et al., 2016). The expression level of vesicular miR-222-3p in epithelial ovarian cancer patients is markedly higher than that in healthy people (Ying et al., 2016). Increased miR-222-3p has the ability to inhibit SOCS3 expression and stimulate the SOCS3/STAT3 pathway in macrophages, thereby inducing TAM-like phenotype macrophages production *in vitro* and *in vivo* (Ying et al., 2016). In addition, hypoxic EVs isolated from tumor cells, including pancreatic cancer and glioma, can generate the M2-like phenotype macrophages (Wang et al., 2018b; Qian et al., 2020). miR-301a-3p is highly enriched in EVs isolated from PANC-1 and BxCP-3 pancreatic cancer cells cultured in hypoxia conditions (Wang et al., 2018b). Mechanically, vesicular miR-301a-3p down-regulates PTEN expression and subsequently activates the PI3Kγ pathway, resulting in increased expression of M2 macrophage marker like CD163 (Wang et al., 2018b). Furthermore, the knockdown of HIF-1a and HIF-2a in pancreatic cancer cells revealed that miR-301a-3p expression level under hypoxia conditions relied on HIF-1a and HIF-2a (Wang et al., 2018b). In glioma, miR-1246 is the most prominently increased content in hypoxic tumor-derived EVs as compared with that in normoxic tumor-derived EVs (Qian et al., 2020). The increased miR-1246 is considered as a key regulator in inducing M2 macrophage polarization, since it can activate the STA3T signaling pathway and suppress the NF-κB signaling pathway (Qian et al., 2020). Notably, the M2 macrophage is a pro-tumor phenotype that promotes tumor cells migration and invasion via facilitating the formation of the immunosuppressive microenvironment. Vesicular lncRNA BCRT1 from breast cancer cells enhances tumor cells migration and invasion (Liang et al., 2020). Injection of breast cancer cells into lncRNA BCRT1-overexpressing mice causes more and larger metastatic lung foci (Liang et al., 2020). In addition, M2 markers (CD206 and MRC-2) expression are shown to up-regulate when macrophages stimulated by EVs derived from lncRNA BCRT1-overexpressing breast cancer cells (Liang et al., 2020). Furthermore, the transfer of Rab22a-NeoF1 fusion protein from osteosarcoma cells to negative tumor cells via EVs contributes to the formation of pre-metastatic niche in osteosarcoma (Zhong et al., 2021). Rab22a-NeoF1 fusion protein recruits bone marrow-derived macrophages and subsequently induces M2 macrophage polarization via its binding partner PYK2 (Zhong et al., 2021). Quantitative real-time PCR (RT-qPCR) analysis showed lncRNA TUC339 was significantly enriched in tumor-derived EVs (Li et al., 2018). Knocking out TUC339 in macrophages resulted in elevated pro-inflammatory cytokines IL-1β and TNF-α (Li et al., 2018). In turn, pro-inflammatory cytokine production decreased in TUC339-overexpressing macrophages (Li et al., 2018). This reveals that the lncRNA TUC339 can serve as a regulator to modulate M2 polarization macrophages.

In addition to mediating transmit miRNA or lncRNA to induce M2 polarization macrophages, EVs have also been shown to deliver miRNA or protein to target cells, thus inducing M1 polarization macrophages. For instance, miR-21 is selectively enriched in EVs isolated from colorectal cancer, which correlates with the increased M1/M2 ratio (Shao et al., 2018). In addition, miR-21 mimic causes increased pro-inflammatory cytokine production in macrophage (Shao et al., 2018). Mechanically, vesicular miR-21 up-regulates IL-6 expression level in macrophages by directly binding to toll-like receptor (TLR) 7, thereby contributing to creating an inflammatory TME (Shao et al., 2018). miR-9 has been found to be markedly enriched in HPV + head and neck squamous cell carcinoma (Tong et al., 2020). In addition, vesicular miR-9 down-regulates PPARδ and subsequently induces M1 polarization macrophages, which consequently leads to enhanced tumor radiosensitivity (Tong et al., 2020). Moreover, EVs isolated from oral squamous cell carcinoma significantly up-regulate the expression levels of pro-inflammatory cytokines (IL-6, IL-1β and TNF-α), while exerting no effect on the expression levels of anti-inflammatory cytokines (IL-10, MRC1 and CCL18) (Xiao et al., 2018). This suggests THBS1 expression is closely correlated with the expression levels of M1 related cytokines. Mechanically, EVs isolated from oral squamous cell carcinoma induce M1 polarization macrophages via stimulating p38, AKT and SAPK/JNK signaling pathways (Xiao et al., 2018).

### T Cells

T cells, including unactivated naive T cells and effector T cells activated by antigen, are the key regulators in the tumor immunity. Helper T cells and cytotoxic T cells are mainly involved in the tumor immunity, while regulatory T cells (Tregs) are mainly involved in tumor immune escape. Nowadays, tumor-derived EVs have been found to act as an immune suppressor to promote recruitment and activation of Tregs in the TME, thereby creating a pro-tumorigenesis environment for tumor progression. For instance, HCC cells-derived EVs have been demonstrated to mediate the delivery of 14-3-3ζ to tumor-infiltrating T cells, which suppresses the anti-tumor effects of T cells (Wang et al., 2018c). Vesicular 14-3-3ζ inhibits the activity and proliferation of peripheral blood T cells, which consequently contributes to deviating the transformation of naive T cells from effector T cells to Tregs (Wang et al., 2018c). In addition, vesicular miR-208b suppresses programmed cell death factor 4 in recipient CD4^+^ T cells and subsequently promotes Tregs proliferation, which consequently accelerates tumor growth in colorectal cancer (Ning et al., 2021). Nasopharyngeal carcinoma cells selectively up-regulate the transcription of CCL20, which serves as a Treg attractor (Mrizak et al., 2015). In addition, nasopharyngeal carcinoma-derived EVs have the ability to recruit Tregs into the TME and induce the transformation of T cells into Tregs, resulting in an enhanced immunosuppression effect in a dose-dependent manner (Mrizak et al., 2015). In addition to mediating Tregs recruitment in TME, tumor-derived EVs have also been shown to regulate the expression of the immune-related genes in Tregs. For example, mRNA profiles analysis revealed that EVs isolated from head and neck squamous cell carcinoma could up-regulate the expression of CD25, CD39, CD73 and CD26 in activated Tregs (Muller et al., 2016). Heat map analysis further found that tumor-derived EVs co-cultured with Tregs would lead to higher expression levels of adenosine-pathway genes and lower expression levels of immunoregulatory genes (Muller et al., 2016). Previous studies revealed that the adenosine pathway was one of the key mechanisms utilized by Tregs to function as an immunosuppressor (Whiteside et al., 2012; Whiteside and Jackson, 2013). This suggests tumor-derived EVs promote the suppression functions of Tregs *via* regulating the expression of adenosine-pathway genes. CD73^+^ γδT cells, serve as the main Tregs subset in breast cancer, are able to mediate immunosuppressive effect in an adenosine-dependent manner (Ni et al., 2020). In the context of breast cancer, the release of lncRNA SNHG16 from EVs is shown to regulate CD73 expression on γδT cells (Ni et al., 2020). Mechanically, vesicular lncRNA SNHG16 stimulates the TGF-β/SMAD5 pathway by targeting the SMAD5 gene, which up-regulates CD73 expression on γδT cells (Ni et al., 2020).

### B Cells

B cells play a key role in humoral immunity on account of their abilities to produce immunoglobulin and present antigens. The regulatory B cells (Bregs) as a subset of B cells are correlated with immunosuppressive response. Similar to T cells, B cells can be induced into Bregs by tumor-derived EVs. For instance, HCC cells-derived EVs can induce TIM-1^+^ Breg with a high expression level of IL-10 (Ye et al., 2018). T cell co-culture with EVs-induced B cell results in decreased TNF-α and IFN-γ production (Ye et al., 2018). Notably, vesicular HMGB1 can activate the TLR-MAPK signaling pathway, which has been found to play a crucial role in inducing the transition of B cells into Bregs (Ye et al., 2018). Further study has demonstrated that blocking TLR or inhibiting MAPK can significantly suppress the Bregs expansion and up-regulate the production of pro-inflammatory cytokines (Ye et al., 2018). In addition, EVs isolated from esophageal squamous cell carcinoma patients significantly enhance the IL-10^+^ Breg production (Mao et al., 2019). Correspondingly, flow cytometry analysis showed that the expression levels of IL-10 and PD-1 in B cells were higher when B cells were co-cultured with tumor-derived EVs (Mao et al., 2019). As EVs commonly function via delivering biomolecular components to target cells, researchers further analyzed the mRNAs and lncRNAs composition in EVs. Results revealed that a total of 947 mRNAs and 175 lncRNAs were down-regulated, while a total of 407 mRNAs and 1,331 lncRNAs were up-regulated in EVs released from esophageal squamous cell carcinoma (Mao et al., 2019). Furthermore, EVs derived from head and neck squamous cell carcinoma directly suppress B cell proliferation and activity (Schroeder et al., 2020). Flow cytometry analysis showed that tumor-derived EVs could inhibit the expression of checkpoint receptors (GITR and BTLA) and CD39 on B cells (Schroeder et al., 2020). Notably, as a B cell activation marker, CD39 regulates adenosine production in many immune cells, thus influencing the immunosuppressive effect of B cells (Saze et al., 2013).

### Immune Cell-Derived EVs

NK cell-derived EVs significantly enhance the apoptosis of aggressive melanoma (Zhu et al., 2017). Meanwhile, the transfer of miR-186 from NK cell to neuroblastoma cell via EVs inhibits tumor growth and reverses immune escape (Neviani et al., 2018). Most TAMs display an M2-like phenotype, and thus M2 macrophages are the predominant macrophage phenotype in the TME. M2 macrophage derive-EVs (M2-EVs) stimulate PI3k/AKT signaling pathway via enriched apolipoprotein E, leading to cytoskeleton remodeling in gastric cancer cells, and ultimately result in enhanced migration (Zheng et al., 2018). M2-EVs also enrich miR-233 under hypoxia conditions. Vesicular miR-233 affords drug resistance to cDDP in epithelial ovarian cancer cells by activating PTEN-PI3k/AKT signaling pathway (Zhu et al., 2019). Moreover, the transfer of miR-21-5p and miR-155-5p *via* EVs from M2 macrophages to colon cancer cells down-regulates BRG1 and consequently promotes tumor metastasis (Lan et al., 2018). By contrast, M1 macrophage-derived EVs potentiate therapeutic efficacy of gemcitabine by improving chemosensitivity of resistant pancreatic cancer cells (Zhao Y. et al., 2021). T cell-derived EVs containing programmed cell death 1 inhibit tumor cell immune escape by triggering programmed death-ligand1internalization (Qiu et al., 2021). In addition, CD4^+^ T cell-derived EVs potentiate vaccine-mediated immune responses by enhancing B cell proliferation and antibodies production (Lu et al., 2019). By contrast, B cell-derived EVs compromise chemotherapeutic effect by attenuating CD8^+^ T cell response (Zhang F. et al., 2019). Thus, inhibition of B cell-derived EVs release contributes to enhanced post-chemotherapeutic T cell responses (Zhang F. et al., 2019).

## Tumor-Derived EVs as Biomarkers in Cancer Diagnosis and Treatment

Due to the lack of ideal biomarkers in the clinic, most cancer patients once diagnosed have been at the advanced stage. Tumor-derived EVs can be released into various body fluids like blood and ascites, which are able to reflect the status of the parental cancer cell. Therefore, tumor-derived EVs are considered as ideal candidates for non-invasive biomarkers in cancer diagnosis. The diversity and specificity of tumor-derived EVs, including miRNA, lncRNA and protein, enable their application in diagnosis (Table 3). For instance, EVs released from acute myeloid leukemia cell selectively enrich let-7a, miR-99b, miR-146a, miR-155, miR-191 and miR-1246 (Hornick et al., 2015). Moreover, RT-qPCR analysis showed that the concentrations of those increased miRNAs were 1000-fold above the cellular level, which may better distinguish acute myeloid leukemia from healthy volunteers with high sensitivity and specificity (Hornick et al., 2015). High levels of circular RNA SETDB1 and miR-31-5P are observed in lung adenocarcinoma patients (Xu et al., 2021; Yu et al., 2021). Serum vesicular circular RNA SETDB1 level is closely correlated with the T stage and lymph node metastasis (Xu et al., 2021). In addition, zinc finger antisense 1 (ZFAS1), belonging to competing endogenous lncRNA, is enriched in the serum EVs of gastric cancer patients (Pan et al., 2017). Highly expressed vesicular ZFAS1 may be related to a higher risk of lymphatic metastasis in gastric cancer patients (Pan et al., 2017). Glypican-1 (GPC1) is specifically up-regulated in tumor-derived EVs, thus detection of serum-derived EVs from pancreatic cancer patients distinguishes healthy individuals and patients with a benign pancreatic cancer from patients with early- and late-stage pancreatic cancer in a GPC1-dependent manner with specificity and sensitivity (Melo et al., 2015). In addition, contactin-1 is selectively elevated in plasma EVs of melanoma cancer patients when compared with EVs of normal volunteers (Pietrowska et al., 2021). This indicates that the detection of these differentially expressed proteins of melanoma cancer-derived EVs may play an essential role in the diagnosis and monitoring of tumors. The expression levels of let-7p-3b, miR-150-3p, miR-145-3p and miR-139-3p in plasma-derived EVs from colon cancer patients are much higher than those in plasma-derived EVs from healthy controls (Min et al., 2019). Moreover, EVs derived miRNAs show a more potent diagnosis efficacy than plasma total miRNAs. Recently, Xia et al. (2020) believe that miR-301a-3p is correlated to gastric cancer development and metastasis. In addition, miR-301a-3p is selectively enriched in serum EVs isolated from gastric cancer with peritoneal metastasis (Xia et al., 2020). Similarly, elevated miR-92a-3p expression level in plasma-derived EVs is related to metastasis of HCC patients (Yang et al., 2020). Also, vesicular lncHILAR expression is markedly higher in renal cancer patients with metastasis than those without metastasis (Hu et al., 2021). Yuan et al. (2021) believe that breast cancer patients with high vesicular miR-21 in serum also have bone metastasis. Furthermore, high vesicular HMGB3 level is observed in nasopharyngeal carcinoma patients, especially those with metastasis (Zhang K. et al., 2021).

**TABLE 3:** Tumor-derived EVs as biomarkers in cancer diagnosis and treatment.

Cargoes	Cancer types	Source of EVs	Applications	References
Let-7a, miR-99b, miR-146a, miR-155, miR-191, miR-1246	Acute myeloid leukemia	Serum	Diagnosis	Hornick et al. (2015)
Circular RNA SETDB1, miR-31-5p	Lung adenocarcinoma	Serum/plasma	Diagnosis	Xu et al. (2021), Yu et al. (2021)
ZFAS1, miR-301a-3p	Gastric cancer	Serum	Diagnosis	Pan et al. (2017), Xia et al. (2020)
GPC1	Pancreatic cancer	Serum	Diagnosis	Melo et al. (2015)
Contactin-1	Melanoma cancer	Plasma	Diagnosis	Pietrowska et al. (2021)
Let-7p-3b, miR-150-3p, miR-145-3p, miR-139-3p	Colon cancer	Plasma	Diagnosis	Min et al. (2019)
miR-92a-3p	HCC	Plasma	Diagnosis	Xia et al. (2020)
LncHILAR	Renal cancer	Plasma	Diagnosis	Hu et al. (2021)
miR-21	Breast cancer	Serum	Diagnosis	Yuan et al. (2021)
HMGB3	Nasopharyngeal carcinoma	Serum	Diagnosis	Zhang et al. (2021a)
TrpC5	Breast cancer	Peripheral blood	Therapy monitoring	Ma et al. (2014)
Annexin A6	Triple-negative breast cancer	Serum	Therapy monitoring	Li et al. (2021)
miR-208b, miR-21-5p	Colorectal cancer	Serum	Therapy monitoring	Ning et al. (2021), He et al. (2021)
S100A4, osteopontin	HCC	Plasma	Prognosis	Sun et al. (2021)
LncRNA-SOX2OT	NSCLC	Peripheral blood	Prognosis	Ni et al. (2021)

In addition to acting as non-invasive biomarkers in cancer diagnosis, EVs have also been shown to serve as “real time” biomarkers during cancer treatment (Table 3). For instance, TrpC5 is a regulator of multidrug transporter P-glycoprotein, which promotes the generation of EVs. The expression level of vesicular TrpC5 in breast cancer patients with low drug sensitivity is significantly higher than that in healthy volunteers (Ma et al., 2014). As a result, detection of TrpC5-bearing EVs in peripheral blood of patients may predict the clinical treatment effect of chemotherapy. Similarly, annexin A6 overexpression relates to poor response to gemcitabine-based chemotherapy (Li et al., 2021). Ning et al. (2021) believe that the elevated miR-208b expression level is associated with oxaliplatin resistance in colorectal cancer patients. In addition, the expression level of vesicular miR-21-5p decreases in colorectal cancer patients after surgical resection (He et al., 2021). Recent studies have found that tumor-derived EVs may be a potential biomarker for cancer prognosis (Table 3). For instance, highly expressed vesicular S100A4 and osteopontin are related to short overall survival rates and disease free survival rates in HCC patients (Sun et al., 2021). Similarly, highly expressed vesicular lncRNA-SOX2OT are also related to short overall survival rates in NSCLC patients (Ni et al., 2021).

To better utilize these biomarkers in clinic, progress in EVs detection should be discussed. The conventional EVs detection methods conclude ultracentrifugation pretreatment and downstream western blotting, ELISA or PCR analysis. However, these techniques are time-consuming and insensitive (Lane et al., 2019). To overcome these limitations, plenty of micro and nano-devices have been exploited for detecting EVs. For example, Lewis et al. (2018) developed an Alternating Current Electrokinetic chip, which could capture and quantify vesicular GPC1 and CD63 within 30 min. Zhang P. et al. (2019) designed a fluorescence-based integrated platform called ExoProfile, which could elucidate the differences between the EVs of ovarian cancer patients and healthy people. Pang et al. (2020) used a surface enhanced Raman scattering method to elucidate the expression level of vesicular PD-L1 between NSCLC patients and healthy people. Recently, Marchisio et al. (2021) used a polychromatic flow cytometry technique to perform the detection of EVs captured by the lipophilic cationic dye. Thakur et al. (2021) presented a localized surface plasmon resonance (LSPR) technique to detect tumor-derived EVs using designed TiN-NH-LSPR biosensor and demonstrated that the label-free LSPR technique can be used for glioblastoma monitoring. Park et al. (2021) proposed a high-throughput electrochemical detection platform called HiMEX, which could distinguish colorectal cancer patients from healthy volunteers with high sensitivity and specificity.

## Conclusion

In recent years, numerous studies of EVs have reported their participation in different stages during cancer progression. The delivery of intercellular information from tumor cells to stromal cells *via* EVs affects the functions and phenotypes of recipient cells, thereby regulating tumor progression. Herein we describe the key findings on how tumor-derived EVs remodel TME to influence tumor progression. In this regard, it is the multiple activators in EVs to create a protumoral or an anti-tumoral soil in the microenvironment. Moreover, such properties also enable their application in cancer diagnosis and treatment as an ideal candidate for non-invasive biomarkers. However, despite significant progress has been made in exploring the role of tumor-derived EVs in TME, many questions remain. Firstly, current studies on tumor-derived EVs use highly heterogeneous cells composed of multiple clones. Therefore, the functions of EVs released from single-cell have yet to be unveiled. Secondly, the enhanced techniques for EVs detection are sensitive and precise, but they also require expensive modification. Thus, highly precise, low-cost and simple techniques for clinical samples detection remain to be exploited. Thirdly, it remains unclear what the main components are at work. This field is in urgent need of more precise characterization of EVs cargo and biology.
